# Long-Term Efficacy and Safety of Guselkumab in Psoriasis Patients Who Failed Anti-IL17: A Two-Year Real-Life Study

**DOI:** 10.3390/jcm13092691

**Published:** 2024-05-03

**Authors:** Matteo Megna, Angelo Ruggiero, Fabrizio Martora, Ylenia Vallone, Gianluca Guerrasio, Luca Potestio

**Affiliations:** Section of Dermatology, Department of Clinical Medicine and Surgery, University of Naples Federico II, Naples, Italy

**Keywords:** guselkumab, psoriasis, real life, anti-IL23, anti-IL17 agents

## Abstract

Guselkumab is the first approved human IgG1λ monoclonal antibody selectively targeting the p19 subunit of interleukin (IL)-23. Despite its effectiveness and safety, which have been widely reported by clinical trials and real-life experiences, data regarding its use on patients who previously failed anti-IL17 are limited or characterized by a reduced follow-up period. These data are essential to guide clinicians in biologic switching, considering that anti-IL23 and anti-IL17 partially share their therapeutic targets, as well as some patients who may have to interrupt treatment with anti-IL17 for loss of efficacy over time or the development of adverse events (AEs). In this context, we performed a retrospective study with the aim of evaluating the long-term use (2 years) of guselkumab in psoriasis patients who previously failed at least one anti-IL17 in a real-life setting, also focusing attention on psoriasis located in difficult-to-treat areas (the scalp, palms or soles, fingernails, genitals). A total of 61 patients (35 male, 57.4%; mean age 57.6 ± 8.8 years) were enrolled. Of these, 30 (49.2%) patients failed secukinumab, 21 (34.4%) failed ixekizumab, 7 (11.5%) failed brodalumab, and 3 (4.9%) failed both secukinumab and ixekizumab. At the baseline, the mean PASI and BSA were 12.8 ± 8.4 and 24.5 ± 26.6, respectively. During week 16, PASI90 and PASI100 responses were achieved by 60.7% and 37.7% of patients, respectively, which continued to improve up to week 104 (PASI90: 73.8%, PASI100: 59.0%). Clinical improvement in difficult-to-treat areas was detected as well. In particular, a slower improvement for fingernails and the palmoplantar region was reported compared to scalp and genital psoriasis at week 16. However, no differences were found following 28 weeks of therapy. Primary and secondary inefficacy were reported by 1 (1.6%) and 5 (8.2%) patients. As regards safety, no severe AEs were collected.

## 1. Introduction

Psoriasis is a chronic inflammatory cutaneous disease affecting up to 3% of the worldwide population [1]. Among its various clinical presentations, plaque psoriasis is the most common (up to 90% of cases), presenting as sharply demarcated erythematous plaques usually involving the extensor surface of elbows and knees, the lower back, scalp, and umbilical areas, but any skin surface can be involved [2]. Several comorbidities can be associated with psoriasis, such as psoriatic arthritis (PsA), inflammatory bowel diseases, metabolic syndrome, type 2 diabetes, cardiovascular disorders, psychiatric diseases, etc. [3]. Moreover, psoriasis may strongly affect patients’ quality of life [3].

In this context, an appropriate and well-designed treatment is needed, not only targeting skin manifestations but psoriatic disease as a whole [4].

Despite the fact that mild forms of this disease are usually well-controlled with topical therapies (mainly the association of calcipotriol and betamethasone), the treatment of moderate-to-severe forms of psoriasis may be challenging [5,6]. Indeed, conventional systemic therapies (acitretin, ciclosporin, methotrexate, and dymethil fumarate) are usually contraindicated for the presence of comorbidities or interrupted for the development of adverse events (AEs). Similarly, the use of phototherapy can be limited by logistic issues [5,6].

Fortunately, the recent introduction of biologic drugs targeting the specific interleukins (ILs) involved in psoriasis pathogenesis revolutionized the therapeutic scenario [5,6]. In particular, 12 different biologics are currently approved for the management of moderate-to-severe plaque psoriasis targeting Tumor Necrosis Factor α (adalimumab, etanercept, infliximab, and certolizumab), IL12/23 (ustekinumab), IL17 (bimekizumab, brodalumab, ixekizumab, and secukinumab), and IL23 (guselkumab, risankizumab, and tildrakizumab). In particular, anti-IL23 are the most recent classes of drugs approved [7,8]. Of these, guselkumab is the first approved human IgG1λ monoclonal antibody selectively targeting the p19 subunit of IL23 [9]. Its effectiveness and safety have been broadly reported by both clinical trials and real-life experiences [10,11,12,13]. However, data regarding its use on psoriasis patients who previously failed anti-IL17 are limited or have a reduced follow-up period. These data are essential to guide clinicians in biologic switching, considering that anti-IL23 and anti-IL17 partially share their therapeutic targets, as well as the fact that some patients may have to interrupt treatment with anti-IL17 for different reasons, including loss of efficacy over time and AEs [1,2]. In this scenario, the aim of our study was to evaluate the effectiveness and safety of the long-term use of guselkumab in psoriasis patients who previously failed an anti-IL17. Moreover, the secondary outcome of our study was to investigate the effectiveness of guselkumab use following anti-IL17 failure in the so-called “difficult-to-treat areas” (scalp, genitals, palms and soles, fingernails). These areas are usually characterized by treatment resistance to conventional topical and systemic treatments, as well as a strong impact on patients’ quality of life [14].

## 2. Material and Methods

A monocentric retrospective study conducted at the Psoriasis Care Centre of Dermatology, University Federico II of Naples, enrolling patients with moderate-to-severe plaque psoriasis who were receiving treatment with guselkumab was performed. In particular, only subjects who previously failed one or more anti-IL17 treatments (brodalumab, ixekizumab, and/or secukinumab) were screened.

Inclusion criteria were as follows: the presence of moderate-to-severe plaque psoriasis assessed by a dermatologist for at least 6 months; guselkumab treatment for psoriasis at a labeled dosage (100 mg at week 0, week 4, and every 8 weeks thereafter) for at least 16 weeks; the previous failure of one or more anti-IL17 treatments (brodalumab, ixekizumab, and/or secukinumab). Exclusion criteria were as follows: patients < 18 years old; concomitant systemic treatment for psoriasis; and erythrodermic psoriasis or generalized pustular psoriasis.

At the baseline, demographic and clinical data (psoriasis duration, body surface area (BSA), Psoriasis Activity Severity Index (PASI), difficult-to-treat areas involving the use of specific BSAs (scalp, palmoplantar, genital), and the Nail Psoriasis Severity Index (NAPSI), comorbidities, previous psoriasis treatments, and the presence of PsA) were collected. Psoriasis severity and AEs were evaluated at each follow-up visit [week 16–28–52–76–108].

Furthermore, at each follow-up visit, routine blood tests (blood count with formula, transaminases, creatinine, azotemia, glycaemia, erythrocyte sedimentation rate, C reactive protein, total cholesterol and triglycerides levels) were assessed to confirm the safety of guselkumab.

A clinical response falling short of PASI75 after 16 weeks was categorized as a primary lack of efficacy. Additionally, the evaluation of insufficient response, defined as <PASI75 after an initial clinical response at 16 weeks, was considered treatment inefficacy, termed secondary inefficacy.

The current study adhered to the principles outlined in the Declaration of Helsinki, and all participating patients provided informed consent prior to their involvement.

### Statistical Analysis

A statistical examination was performed to assess the significance of our results. Globally, descriptive statistics were used to present clinical and demographic data. In particular, these data were presented as the mean ± standard deviation in cases of continuous data, using the number and proportion (%) for categorical ones. Statistical analysis with GraphPad Prism 8.0 (GraphPad Software Inc., La Jolla, CA, USA) was used to evaluate the statistical significance of clinical response, considering statistical significance at a *p*-value < 0.05.

In particular, Student’s *t*-test was used to assess the significance of clinical improvement at the different timepoints of treatment, compared with the baseline. The effectiveness data underwent analysis utilizing the last observation carried forward approach. In instances where a patient discontinued participation in the study, the last recorded value was extended forward and utilized until the conclusion of the treatment period.

## 3. Results

A total of 61 patients (35 male, 57.4%; mean age 57.6 ± 8.8 years; mean psoriasis duration: 23.4 ± 10.1 years) were enrolled. Of these, 27 (44.3%) were also affected by PsA. Clinical and demographic features are summarized in Table 1.

Regarding comorbidities, hypertension was reported the most frequently (*n* = 22, 36.1%), followed by dyslipidaemia (*n* = 20, 32.8%) and obesity (*n* = 17, 27.9%).

Moreover, all the patients received at least one conventional systemic treatment or small molecule, with methotrexate as the most common (*n* = 44, 72.1%), followed by cyclosporine (*n* = 24, 39.3%) and narrow band UVB phototherapy (*n* = 11, 18.0%) (Table 1).

Similarly, all the patients failed at least one treatment with a biologic (Table 1).

Regarding previous anti-IL17, 30 (49.2%) patients failed secukinumab, 21 (34.4%) ixekizumab, 7 (11.5%) failed brodalumab, and 3 (4.9%) failed both secukinumab and ixekizumab.

Among the patients failing secukinumab (*n* = 30, 49.2%), 24 (80.0%) discontinued treatment for secondary inefficacy and 4 (13.3%) for primary inefficacy, respectively. Similarly, among the patients receiving ixekizumab (*n* = 21, 34.4%), 15 (71.4%) and 4 (19.0%) discontinued the biologic for primary or secondary inefficacy. Only treatment discontinuation for secondary inefficacy (*n* = 6, 85.7%) or AEs (*n* = 1, 14.3%) were collected among the seven patients previously treated with brodalumab.

Of interest, two (6.7%) and two (9.5%) patients receiving secukinumab and ixekizumab interrupted the treatment for the development of AEs, respectively. Finally, the three patients that reported previously being treated with both secukinumab and ixekizumab discontinued both treatments for secondary inefficacy.

Guselkumab was administered as a second-line, third-line, fourth-line, and fifth-line of biologic treatment in 6 (9.8%), 29 (47.5%), 21 (34.4%), and 5 (8.2%) subjects, respectively.

Clinical evaluation at the baseline showed a mean PASI of 12.8 ± 8.4 and a BSA of 24.5 ± 26.6, respectively.

Both scores starting to improve after week 16 (PASI: 3.3 ± 2.9, BSA: 4.2 ± 3.5, *p* < 0.0001 for both), continuing to improve and maintain clinical response at week 52 (PASI: 1.4 ± 1.8, BSA: 2.6 ± 1.9, *p* < 0.0001 for both), up to week 108 (PASI: 1.0 ± 1.4, BSA: 1.9 ± 1.6, *p* < 0.0001 for both) (Table 2).

At week 16, PASI90 and PASI100 responses were achieved by 60.7% (*n* = 37) and 37.7% (*n* = 23) of patients, respectively. These results were reached by 42 (68.9%) and 28 (45.9%) patients at week 28, by 44 (72.1%) and 32 (52.5%) subjects at week 52, up to 45 (73.8%) and 36 (59.0%) patients after 2 years of treatment with guselkumab.

A sub-analysis of our patients showed that there were no differences in therapeutic outcomes when comparing the previously failed anti-IL17 treatment (secukinumab. ixekizumab, or brodalumab).

**Figure 1:**
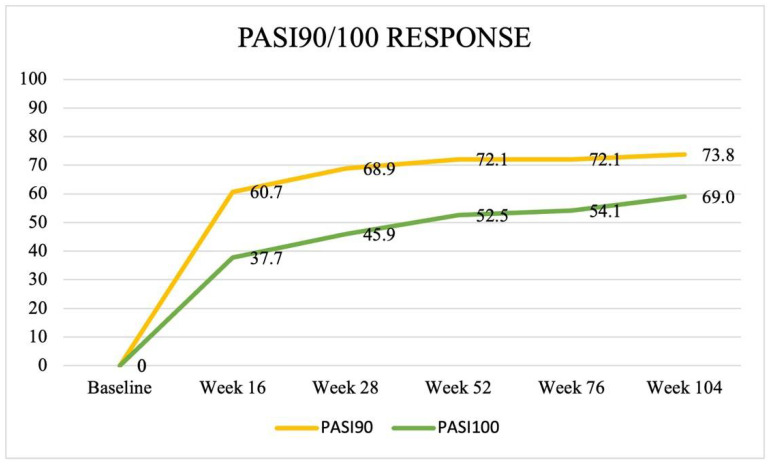
Percentage of patients achieving PASI90 and PASI100 response at week 16, week 28, week 52, week 76, and week 108.

Regarding difficult-to-treat areas, the scalp, palmoplantar, genital, and fingernails were involved in 28 (45.9%), 12 (19.7%), 7 (11.5%), and 23 (37.7%) of patients, respectively. Clinical improvement is summarized in Table 2 and Figure 1.

Observing psoriasis improvement in difficult-to-treat areas, we noted a reduction of 77.8%, 51.8%, and 65.6% in scalp psoriasis, palm or sole psoriasis, and genital psoriasis at week 16. Moreover, psoriasis continued to improve in these areas, reaching a reduction of 98.5%, 93.2%, and 98.2% in the scalp, palms or soles, and genital psoriasis after 2 years of treatment. Regarding nail involvement, at the baseline, NAPSI was 13.1, reducing to 5.9 at week 16 (−55.0%), to 1.6 (−87.8%) at week 52, and up to 1.1 at week 104 (−91.6%).

**Figure 2:**
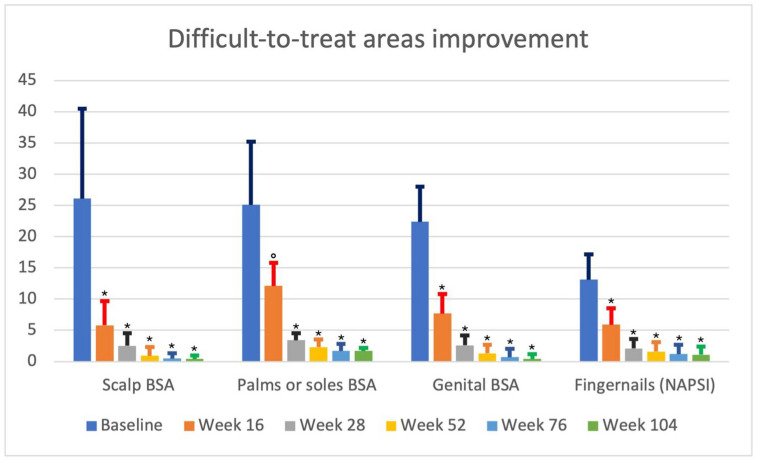
Difficult-to-treat areas improving from the baseline to week 16, week 28, week 52, week 76, and week 108. °: *p* < 0.001 compared with the baseline; *: *p* < 0.0001 compared with the baseline.

Of interest, a slower improvement for the fingernails and palmoplantar region was collected compared with scalp and genital psoriasis (Table 2 and Figure 2). However, we noted that this improvement was similar following 28 weeks of therapy.

As regards treatment discontinuation for inefficacy, 1 (1.6%) patient interrupted guselkumab for primary inefficacy, whereas 5 (8.2%) subjects discontinued the drug for secondary inefficacy.

As regards the safety profile of guselkumab, no severe AEs were detected.

## 4. Discussion

Recent knowledge on psoriasis pathogenesis, particularly on the role of the IL23/17 axis, has led to the development of new effective and safe drugs [15,16]. To date, there are 12 biologic drugs approved for the management of moderate-to-severe plaque psoriasis [15]. Among these, anti-IL23 is the latest class of biologics approved, with guselkumab as the first licensed drug belonging to this class. To date, the efficacy of guselkumab has been first investigated by clinical trials and subsequently confirmed by real-life experiences.

Of note, data from real life are needed to offer patients a tailored approach, especially considering that data from real world are essential as patients in clinical trials are not representative of the daily clinical practice population since real-life subjects might vary, and the specific inclusion and exclusion criteria used in clinical trials reduce the interindividual clinical variability [17].

In this context, studies on the effectiveness of guselkumab following the failure of an anti-IL17 treatment in psoriasis patients are needed to guide clinicians in biologic switching, investigating if a previous failure of a drug acting on the IL23/17 axis may reduce the effectiveness of a second biologic active on the same pathway [3]. In this scenario, we performed a long-term, retrospective, monocentric study with the aim of evaluating the long-term (2 years) effectiveness and safety of guselkumab in psoriasis patients who previously failed at least one anti-IL17 treatment.

A total of 61 subjects were enrolled, with a mean PASI and mean BSA at the baseline of 12.8 ± 8.4 and 24.5 ± 26.6, respectively.

A statistically significant reduction in both PASI and BSA was observed from week 16 (PASI: 3.3 ± 2.9, BSA: 4.2 ± 3.5, *p* < 0.0001 for both), with 60.7% and 37.7% of patients reaching PASI90 and PASI100 response at this timepoint. Moreover, clinical scores continued to improve at week 52 (PASI: 1.4 ± 1.8, BSA: 2.6 ± 1.9, *p* < 0.0001 for both) up to week 104 (PASI: 1.0 ± 1.4, BSA: 1.9 ± 1.6, *p* < 0.0001 for both), with PASI90 and PASI100 response achieved by 72.1% and 52.5%, and by 73.8% and 59.0% of subjects following 1 year and 2 years of treatment with guselkumab, respectively.

Furthermore, guselkumab was shown to also be effective in difficult-to-treat areas. Of interest, scalp and genital psoriasis seemed to improve faster compared with the fingernails and palmoplantar psoriasis. However, the improvement of each difficult-to-treat area was similar following 28 weeks of therapy.

Only 6 treatment discontinuations were reported: 1 (1.6%) for primary inefficacy and 5 (8.2%) for secondary inefficacy.

Finally, no severe AEs were collected.

To sum up, a significant and sustained efficacy of guselkumab was reported up to week 104 despite anti-IL17 failure, with an excellent safety profile.

Globally, our results are in line with the published literature.

Megna et al. reported a larger real-world experience, showing a statistically significant improvement of PASI and BSA at each follow-up from the baseline (PASI: 13.9 ± 8.1; BSA: 24.3 ± 19.6) to week 52 (PASI: 0.9 ± 0.7; BSA: 1.3 ± 1.4), without serious AEs and only 3 (6.8%) discontinuations for secondary inefficacy [18]. In particular, 44 subjects were enrolled. Despite the percentage of PASI90 and PASI100 response observed at week 12, they were similar to ours at week 16 (PASI90: 61.4% vs. 60.7%; PASI100 31.8% vs. 37.7%), and a reduced number of PASI90 and PASI100 achievement were reached at week 52 in our cohort (PASI90: 79.5% vs. 72.1%; PASI100: 64.8% vs. 52.5%) [18]. In probability, the different sizes of the two cohorts may explain these differences.

Similarly, Ruiz-Villaverde et al. also confirmed that the use of guselkumab was not impacted by previous biologics, with a treatment survival of 92.7% of patients at week 130 among subjects previously receiving an anti-IL17 (*n* = 29), compared with 100% and 92.1% of patients previously treated with anti-TNFα, (*n* = 29) and ustekinumab (*n* = 45) [19]. On the contrary, Hung et al. reported that patients previously receiving anti-IL17, rather than anti-TNF and IL-12/23, had reduced PASI improvement compared with biologic-naïve patients at week 12, week 20, and week 28, suggesting that biologic exposure, particularly anti-IL17, may reduce guselkumab effectiveness [20]. In our opinion, the reduced follow-up period (36 weeks) and the reduced cohort (33 patients) may explain these results, which were not confirmed in our study.

Ruggiero et al. reported the results of a 52-week retrospective study enrolling 13 patients previously treated with ustekinumab (*n* = 6, 46.1%) and/or an anti-IL17 (*n* = 9, 69.2%). PASI and BSA were significantly reduced from the baseline (PASI: 13.2 ± 6.8, BSA: 22.3 ± 10.5) to week 4 (PASI: 5.9 ± 2.8, BSA: 14.2 ± 7.5; *p* < 0.01 for both), continuing to improve up to week 52 (PASI: 0.5 ± 0.7, BSA: 0.8 ± 1.1; *p* < 0.001 for both) [21]. Specifically, the authors did not report statistically significant differences between patients previously treated with anti-IL12/23 compared to anti-IL-17 or both [21]. Finally, no serious AEs were reported, and only one patient previously treated with ustekinumab and secukinumab discontinued guselkumab for secondary inefficacy [21]. In line with our data, previous treatment with anti-IL17 does not seem to affect the efficacy of guselkumab.

Similarly, Giordano et al. suggested that switching from anti-IL17 to guselkumab was effective and safe in their 52-week study, enrolling 14 out of a total of 48 (29.2%) subjects previously treated with anti-IL17 [22]. However, three patients discontinued treatment for inefficacy [22].

A significant improvement in psoriasis after 12 weeks of guselkumab treatment following anti-IL17 treatment were also reported by Bonifati et al. [23]. In particular, among the 12 enrolled patients, 9 were switched to guselkumab and 3 to risankizumab [23]. ThePASI, Dermatology Life Quality Index, Physician Global Assessment, and Visual Analogic Scale for Itch significantly decreased throughout the 24 weeks of therapy with guselkumab or risankizumab [23]. These reductions were already significant after 12 weeks (Wilcoxon test < 0.0001 for all) [23].

Finally, the effectiveness and safety of guselkumab was also suggested by two short-term studies enrolling 180 and 55 patients [24,25]. In particular, in the real-life experience reported by Fougerousse et al. with the aim of investigating the effectiveness and short-term (16 weeks) tolerance of guselkumab for psoriasis under real-life conditions, only 39 out of 180 subjects had previously failed anti-IL17 treatment [24]. Despite guselkumab being shown to be effective (PASI90: 50.6% and PASI100: 38.3%) and safe (no serious AEs collected), the specific data for patients who previously failed anti-IL17 were not discussed [24].

Similarly, despite the fact that anti-IL17 failed in 34 cases among the 55 patients reported in the real-life experience by Rodriguez Fernandez-Freire, and the effectiveness and safety of guselkumab was reported, specific data for patients previously failing an anti-IL17 were not investigated [25].

To sum up, our experience in this study with a larger cohort and follow-up period specifically investigated the effectiveness of guselkumab after anti-IL17 failure.

Moreover, our study was the first to specifically investigate the efficacy of guselkumab in patients who previously discontinued anti-IL17 affected by psoriasis that was also located in difficult-to-treat areas.

Our data confirm previous results, which highlight guselkumab as a valuable option in patients with moderate-to-severe psoriasis and unresponsive to biologics targeting IL17, suggesting the efficacy and safety of this drug in the long term. Furthermore, guselkumab was shown to be an effective weapon for the management of psoriasis located in difficult-to-treat areas.

## 5. Strengths and Limitations

The main strengths of our study are the accuracy of the data, the consideration of difficult-to-treat areas, the long follow-up period, and the homogeneity of clinical evaluation. However, the limitations of this study should be discussed. The main limitations are the reduced cohort and the retrospective design of the study. Moreover, this study lacks a control group. Indeed, only 3 (4.9%) patients underwent an intraclass switch from one anti-IL-17 biologic to another (ixekizumab + secukinumab). Future research should compare the durability of treatments between anti-IL-23 biologics and a second anti-IL-17 biologic in psoriasis patients who did not respond to their first anti-IL-17 treatment. Finally, it should be noted that there were no patients who failed bimekizumab since it was recently approved.

## 6. Conclusions

Our study confirmed the efficacy and safety of guselkumab in the long term, suggesting that the previous failure of anti-IL17 does not seem to affect the effectiveness of guselkumab. Certainly, further studies are needed.

## Figures and Tables

**Table 1:** Patients’ features at baseline (week 0). Nb-UVB (Narrowband–ultraviolet B). PASI: Psoriasis Activity Severity Index. BSA: body surface area. NAPSI: Nail Psoriasis Severity Index (NAPSI).

**Number of Patients**	61
**Sex:**	
Male	35 (57.4%)
Female	26 (42.6%)
**Mean age** (*years*)	57.6 ± 8.8
**Mean duration of psoriasis** (*years*)	23.4 ± 10.1
**Psoriatic Arthritis**	27 (44.3%)
**Difficult-to-treat areas involvement**	
Scalp	28 (45.9%)
Fingernails	23 (37.7%)
Palms or soles	12 (19.7%)
Genital	7 (11.5%)
**Comorbidities:**	
Hypertension	22 (36.1%)
Dyslipidemia	20 (32.8%)
Obesity	17 (27.9%)
Diabetes	10 (16.4%)
Depression	7 (11.5%)
Hypothyroidism	3 (4.9%)
Cardiopathy	3 (4.9%)
**Previous systemic treatments (conventional and small molecules):**	
Methotrexate	44 (72.1%)
Cyclosporine	24 (39.3%)
Nb-UVB phototherapy	11 (18.0%)
Acitretin	9 (14.8%)
Apremilast	5 (8.2%)
**Previous biologic treatments:**	
**Anti-TNFα**	
Adalimumab	33 (54.1%)
Etanercept	12 (19.7%)
Infliximab	5 (8.2%)
Golimumab	4 (6.6%)
Certolizumab	6 (9.8%)
**Anti-IL12/23**	24 (39.3%)
**Anti-IL17**	
Secukinumab	30 (49.2%)
Ixekizumab	21 (34.4%)
Brodalumab	7 (11.5%)
Ixekizumab + secukinumab	3 (4.9%)

**Table 2:** Psoriasis assessment at the baseline, week 16, week 28, week 52, week 76, and week 108. PASI: Psoriasis Activity Severity Index. BSA: body surface area. NAPSI: Nail Psoriasis Severity Index (NAPSI).

	Baseline		Week 16		Week 28		Week 52		Week 76		Week 108	
**Mean PASI**	12.8 ± 8.4		3.3 ± 2.9 (*p* < 0.0001)		1.8 ± 2.1 (*p* < 0.0001)		1.4 ± 1.8 (*p* < 0.0001)		1.2 ± 1.6 (*p* < 0.0001)		1.0 ± 1.4 (*p* < 0.0001)	
**Mean BSA**	24.5 ± 16.6		4.2 ± 3.5 (*p* < 0.0001)		3.5 ± 2.1 (*p* < 0.0001)		2.6 ± 1.9 (*p* < 0.0001)		2.2 ± 1.8 (*p* < 0.0001)		1.9 ± 1.6 (*p* < 0.0001)	
**PASI90**	NA		37 (60.7%)		42 (68.9%)		44 (72.1%)		44 (72.1%)		45 (73.8%)	
**PASI100**	NA		23 (37.7%)		28 (45.9%)		32 (52.5%)		33 (54.1%)		36 (59.0%)	
**Difficult-to-treat areas**				**Variation from T0**		**Variation from T0**		**Variation from T0**		**Variation from T0**		**Variation from T0**
Scalp BSA	26.1%	/	5.8%	−77.8%	2.5%	−90.0%	0.9%	−96.6%	0.5%	−98.1%	0.4%	−98.5%
Palms or soles BSA	25.1%	/	12.1%	−51.8%	3.4%	−86.5%	2.3%	−90.8%	1.7%	−93.2%	1.7%	−93.2%
Genital BSA	22.4%	/	7.7%	−65.6%	2.6%	−88.4%	1.3%	−94.2%	0.7%	−96.9%	0.4%	−98.2%
NAPSI	13.1	/	5.9	−55.0%	2.1	−84.0%	1.6	−87.8%	1.2	−90.8%	1.1	−91.6%

## Data Availability

The data that support the findings of this study are available on request from the corresponding author.
